# Aberrant right subclavian artery with atrial septal defect: Simultaneous repair via median sternotomy

**DOI:** 10.1016/j.ijscr.2020.01.002

**Published:** 2020-01-16

**Authors:** Hina Inam, Abdul Ahad Sohail, Narmeen Asif, Waris Ahmad

**Affiliations:** Aga Khan University Hospital, Pakistan

**Keywords:** Aberrant right subclavian artery, Median sternotomy

## Abstract

**Introduction:**

Aberrant right subclavian artery, also known as arteria lusoria, is one of the rarer congenital vascular malformations of the left sided aortic arch which arises distal to the left subclavian artery, following a retroesophageal course to the right side causing compression of the trachea or esophagus and resulting in a variety of symptoms most commonly dysphagia.

**Presentation of case:**

We report a case of a two and a half year old child who was diagnosed as having as having an aberrant right subclavian artery with an ostium secundum atrial septal defect and underwent simultaneous repair of both conditions via a median sternotomy.

**Discussion:**

Aberrant right subclavian artery (ARSA) has a prevalence of 1.8 % and most commonly presents in fourth or fifth decade of life. Various surgical approaches have been described to repair this anomaly over the past years, each with its own limitations and complications. However, the median sternotomy approach provides adequate exposure during division and re-implantation of the vessel and simultaneously allows repair of any intra-cardiac defect.

**Conclusion:**

We believe that the best exposure for the correction of aberrant right subclavian artery is via a median sternotomy, especially in pediatric patients associated with other cardiac anomalies.

## Highlights

• Aberrant Right Subclavian artery is also known as Arteria Lusoria.
• Rarer congenital malformations of the aortic arch.
• Causes compression of the trachea or esophagus causing dysphagia.
• May occur simultaneously with an ostium secundum atrial septal defect.
• Both conditions repaired simultaneously via Median Sternotomy.

## 1 Introduction

Amongst the various embryological defects of the arch of aorta, one of the frequent defects is an aberrant right subclavian artery (ARSA) with a reported prevalence of only 0.16 %–4.4 % in the general population [1]. It has an incidence of up to 3 % in cases of congenital cardiac defects, presenting most commonly in patients of Down syndrome [2,3]. It’s discovery dates back to 1735 when it was first described by Hunauld [1,4] with its first successful surgical treatment described by Gross in 1946 by simple ligation and division [5].

Normal anatomy of the aortic arch includes a brachiocephalic artery which divides into a right subclavian and right common carotid artery, then the left common carotid and left subclavian artery. However if an ARSA is present than there is no brachiocephalic artery rather a right common carotid, left common carotid and a left subclavian artery can be visualized, while the ARSA is seen arising after the left subclavian artery also known as arteria lusoria This than passes most frequently behind the esophagus and doing so it compresses the esophagus leading to dysphagia more commonly known as dysphagia lusoria [6,7].

Here we present a rare case of a two and a half year old girl who presented with an anomalous origin of the right subclavian artery, associated with an ostium secundum atrial septal defect (ASD). The patient underwent repair of both the anomalies through median sternotomy, with implantation of the right subclavian artery into the right common carotid artery. We believe that median sternotomy is the best surgical approach for the management of these lesions in a simultaneous repair, as described below. This case has been reported in line with SCARE criteria [8].

## 2 Presentation of case

**Fig. 1:**
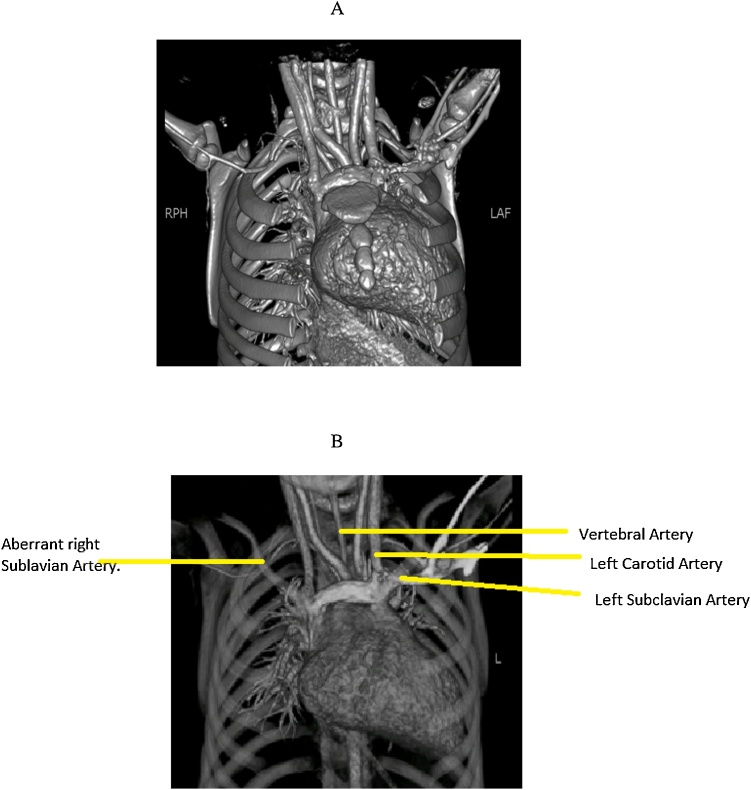
A 3-D reconstruction computed tomography scan showing an aberrant right subclavian artery arising from an undersurface of the arch of the aorta. (A) Anterior view. (B) Posterior view.

A two and a half year old baby girl with dysmorphic features and a history of an atrial septal defect and cleft palate presented to the cardiothoracic clinic with history of recurrent respiratory tract infections, vomiting and aspiration pneumonia. She was evaluated and on computed tomography scan she was found to have a left-sided aorta. The first branch from the arch of the aorta was seen to be the right common carotid artery, second was the left common carotid artery, the third branch was left vertebral artery and fourth one was left subclavian artery as seen in Fig. 1. From the under surface of arch was arising the aberrant right subclavian artery, which was traversing behind the esophagus and trachea to reach the right side of the neck. And in doing so was causing posterior compression of the esophagus. She was also found to have a large secundum ASD which approximately measured 14 mm on echocardiography.

### 2.1 Operative procedure

**Fig. 2:**
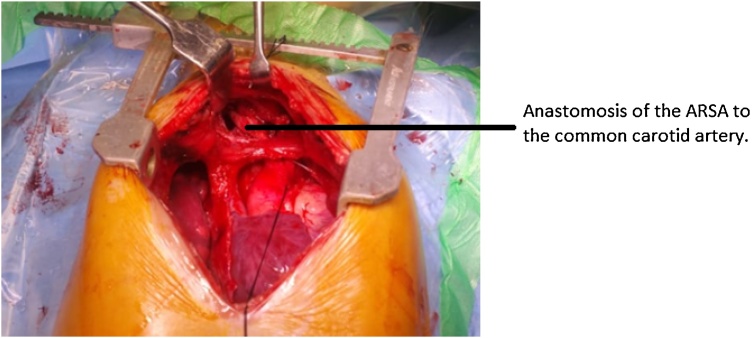
Intra-operative picture showing anastomosis of the right subclavian artery to the right common carotid artery.

A pre and post arterial lines were inserted and after the patient had been prepped and draped under general anesthesia, a median sternotomy was performed. Followed by a thymectomy and pericardectomy.The right pleura was then opened and right subclavian artery was identified and dissected. The rest of the aortic arch was then dissected. Arteria lusoria was identified and isolated. Dissection was further done behind the esophagus and trachea. Loss of the right radial artery trace on clamping the aberrant vessel confirmed that the vessel was definitely the right subclavian artery. Heparin at a dose of 1 mg/kg intravenously was given achieving an activated clotting time (ACT) of greater than 250. The artery was carefully divided at its site of origin and the aortic end was over sewn in two layers with continuous polypropylene sutures. The artery was next brought to the right side from the front of trachea and then anastomosed end side to the right common carotid artery, after carefully evaluating that there was no torsion and twisting of the mobilized vessel (Fig. 2). Remainder of the heparin was given followed by a standard aorto bicaval cannulation and bypass commenced. Cross clamp was applied and del Nido cardioplegia was administered. Pericardial patch closure of the ostium secundum ASD was performed in a routine manner. The patient had an uneventful postoperative course and was discharged on day five from the hospital. The patient had no complaints of dysphagia or regurgitation of food on discharge

## 3 Discussion

Aberrant right subclavian artery (ARSA) has a prevalence of 1.8 % [7] and in 0.4–1.8 % individuals it may arise as a separate branch from the arch of aorta distal to the left subclavian artery rather than a branch of brachiocephalic artery [9,10]. It is present in 1–2 % or normal individual however an increase incidence is seen in individuals suffering from trisomy 21 [11]. It most commonly presents in fourth or fifth decade of life with the average age of presentation at 48 years [5,12]. However in our case the patient was a small child and she was treated due to significant symptoms arising due to the anomaly.

This condition usually remains asymptomatic with symptoms appearing in only about 10–33 % of cases: hence the reason of delayed presentation. Dysphagia being the common symptom followed by dyspnea, cough, recurrent respiratory tract infections and stridor [1,13]. The ARSA compressing the trachea and/or esophagus depending upon its course is responsible for various presentations seen among these patients. [10]. Most commonly retroesophageal ARSA is seen (80–84 %) followed by a course between trachea and esophagus (12.7–15 %) and very rarely pretracheal ARSA (4.2–5 %) may also be seen [1].

Hugo Zapata in his study found ARSA in 128 patients, amongst which 117 patients were with congenital heart disease. These 117 patients with congenital heart disease could be classified according to cyanosis-- present in 72 (62 %) cases, no cyanosis present in 40 (34 %) cases, and a variable cyanosis in five (4 %) cases. The 117 cardiac anomalies were grouped into five general categories. These were cono-truncal anomalies (45 cases, 38 %), septal defect (33 cases, 28 %), left heart obstructions (24 cases, 21 %), right heart anomalies (six cases, 5 %), and other (nine cases, 8 %) [14].

Patients with this rare anomaly having symptoms of tracheal and esophageal compression and those with aneurismal dilatation warrant early surgical intervention [12,13]. There have been different surgical approaches defined by different groups over a period of time regarding the optimal surgical technique to repair this anomaly, each with its own advantages and limitations. These include left thoracotomy in isolation or combined with right supraclavicular incision, right posterolateral thoracotomy and median sternotomy [5,12,15]. Using a right thoracotomy approach may lead to persistent dysphagia from the residual stump of the ARSA if the artery is not divided close enough to the origin [5]. Left thoracotomy provides easy access for ligation and division of ARSA especially when it has a wide base on the proximal descending aorta, however it gives limited exposure and becomes technically challenging during the re-implantation of the aberrant vessel and may need to be combined with right supraclavicular approach [5,12]. However, the median sternotomy approach provides adequate exposure during division and re-implantation of the vessel and simultaneously allows repair of any intra-cardiac defect such as ASD as was done in our case. Hence our case describes a method of surgical approach for a rare congenital vascular malformation that has been not been used in the past very often and an intra-cardiac defect was repaired simultaneously via this approach.

## 4 Conclusion

We believe that the best exposure for the correction of aberrant right subclavian artery is via a median sternotomy, especially in pediatric patients associated with other cardiac anomalies. This approach enables optimal exposure and mobilization to the distal right subclavian artery and end to end anastomosis. It also allows for defects like atrial septal defect or a ventricular septal defect to be closed in the same setting.

## Sources of funding

None.

## Ethical approval

I am submitting a Case Report which is exempted from ethical approval in Aga Khan University Hospital.

## Consent

Yes the consent was taken from the patient’s guardian as the patient in our case is a child.

## Author’s contribution

Hina Inam: Study concept, design, literature search, writing of paper, final review, critical review.

Abdul Ahad Sohail: Study design, writing of paper and drafting of paper and review of paper.

Narmeen Asif: Study concept, design, literature review, writing of paper and review.

Waris Ahmad: Study concept and design, literature review, finalising of paper and critical review.

## Registration of research studies

Not a study involving human studies. A case that was observed has been reported.

## Guarantor

Hina Inam.

Abdul Ahad Sohail.

Narmeen Asif.

Waris Ahmad.

## Provenance and peer review

Not commissioned, externally peer-reviewed.

## Declaration of Competing Interest

No Conflicts of Interest.
